# Fire enhances changes in phosphorus (P) dynamics determining potential post-fire soil recovery in Mediterranean woodlands

**DOI:** 10.1038/s41598-024-72361-8

**Published:** 2024-09-17

**Authors:** P. Souza-Alonso, S. A. Prats, A. Merino, N. Guiomar, M. Guijarro, J. Madrigal

**Affiliations:** 1 Department of Soil Science and Agricultural Chemistry, Higher Polytechnic Engineering School, University of Santiago de Compostela, 27002 Lugo, Spain; 2 MED – Mediterranean Institute for Agriculture, Environment and Development & CHANGE – Global Change and Sustainability Institute, Instituto de Investigação e Formação Avançada, Universidade de Évora, Pólo da Mitra, Ap. 94, 7006-554 Évora, Portugal; 3 Instituto de Ciencias Forestales, ICIFOR-INIA (CSIC), Ctra. Coruña Km 7.5, 28040 Madrid, Spain; 4 ETSI Montes, Forestal y del Medio Natural, Universidad Politécnica de Madrid (UPM), Ramiro de Maeztu s/n, 28040 Madrid, Spain; 5 CHANGE – Global Change and Sustainability Institute, IIFA – Institute for Advanced Studies and Research, EaRSLab – Earth Remote Sensing Laboratory, Universidade de Évora, Évora, Portugal; 6 Misión Biológica de Galicia – Consejo Superior de Investigaciones Científicas (MBG-CSIC), Salcedo Pontevedra, España

**Keywords:** Fire impact, Prescribed burnings, Soil organic layer, Organic–inorganic P, ^31^P-NMR, P availability, Environmental sciences, Geochemistry, Element cycles, Natural hazards

## Abstract

Soil phosphorus (P), which is essential for ecosystem functioning, undergoes notable changes after fire. However, the extent to which fire characteristics affect P dynamics remains largely unknown. This study investigated the impact of type of fire (prescribed burning and natural wildfires) of different levels of severity on P dynamics in Mediterranean soils. Soil P concentrations in the organic layers were strongly affected by fire severity but not fire type. Low severity fire did not have any observable effect, while moderate fire increased soil P levels by 62% and high severity decreased soil P concentration by 19%. After one year, the soil P concentration remained unchanged in the low severity fires, while rather complex recovery was observed after moderate and high severity fires. In the mineral layers, P concentration was reduced (by 25%) immediately after the fires and maintained for one year (at 42%). ^31^P-NMR spectroscopy revealed almost complete post-fire mineralization of organic P forms (mono- and diesters), large increases in inorganic orthophosphate and a decrease in the organic:inorganic P ratio (*P*_*o*_*:P*_*i*_). After one year, di-esters and orthophosphate recovered to pre-fire levels at all sites, except those where parent material composition (high pH and Fe concentration) had an enduring effect on orthophosphate retention, and thus, on the total soil P. We showed that fire severity and soil pH (and hence, soil mineralogy) played an essential role in soil P dynamics. These findings are important for reliable assessment of the effects of fire on soil P conservation and for improving the understanding the impact of prescribed burning.

## Introduction

Fire is a natural, pervasive force that shapes ecosystems and plays a key role in their functioning and configuration^1^, influencing vegetation dynamics, nutrient cycling and soil properties^2^. Despite the importance of understanding the ecological implications, fire generates much controversy and strong debate. The negative perception of fire, especially in non-adapted systems, contrasts with the use of prescribed burning (PB) to prevent large wildfires^3^. Prescribed burning is gradually being adopted in Mediterranean countries as an important tool for fuel management, to limit the risk and to reduce impacts and negative consequences of large, severe wildfires^3,4^. Although the implementation of PB remains limited, different technical studies have assessed the effects on overland flow^5^ and soil erosion^6^ as well as the short- and long-term dynamics of soil properties, including soil organic matter (SOM)^4,7–10^ and soil phosphorus^11^.

The impacts of fire on soils are directly related to fire intensity, soil burn severity (SBS) and meteorological conditions. Prescribed burning, involving lower fire intensity, has low to moderate effects on soil properties^4^. The use of visual indicators such as the SBS index^12^ serves to describe and integrate fire effects at the soil level. Low SBS levels after PB have slight effects on run-off and soil erosion^13^, variable effects on soil carbon (C), nitrogen (N) and phosphorus (P)^6,14^ and limited impacts on microbial community activity and composition^15^.

Phosphorus is one of the most limiting nutrients for plant growth, affecting the productivity of many agrosystems and natural ecosystems^16,17^ and representing a major constraint to global food security and forestry production^18–20^. This limitation is particularly important in highly weathered soils^21^ or soils where P is immobilized by microorganisms, adsorbed or precipitated by physicochemical conditions and soil properties^22,23^.

Except in arid and semiarid soils, the largest fraction of soil P in forest soils (a fraction that varies across different systems) is represented by organic forms (P_o_), which can be mineralized to bioavailable inorganic P. Most P_o_ in the soil is associated with SOM, but its chemical nature is complex and not fully known^24^. Among the organic P forms, the P-monoesters dominate over P-diesters on the forest floor^25–28^. P availability is determined by the distribution of inorganic and organic P forms, and it is generally essential for plant uptake and P cycling. P can only be taken up by plants as inorganic P (mainly orthophosphate), which must be continually replenished from the solid phase as it is present at low concentrations in the soil solution^29^. The accumulation and consumption of P are governed by intricate soil–plant-microbial P cycling interactions, which are, in turn, highly dependent on prevailing environmental and management conditions^30^. Although not fully understood, the importance of P in terms of ecosystem nutrition is such that it has been suggested that plants and soil microorganisms may shape and transform soils on the basis of P demands^31^.

The cycling and movement of the different soil elements, including P, are closely associated with SOM dynamics, which is greatly affected in post-fire scenarios^32,33^. The availability of labile C affects the immobilization and release of P and, therefore, the distribution of P forms^29,34^. Soil alkalinization and P mineralization to more accessible (inorganic) forms also lead to rapid P release and availability. Short-term P enrichment induced after fire is not maintained and solubility can rapidly decline as inorganic P (mainly orthophosphate) binds to Al, Fe and Mn oxides through chemisorption in acid soils, while in neutral or alkaline soils it binds to Ca-minerals or precipitates as discrete Ca-phosphate^33^. Large changes in P availability, such as those observed during post-fire mineralization, also accelerate irreversible P fixation or loss (associated with ash) via wind or water erosion^35^.

**Fig. 1:**
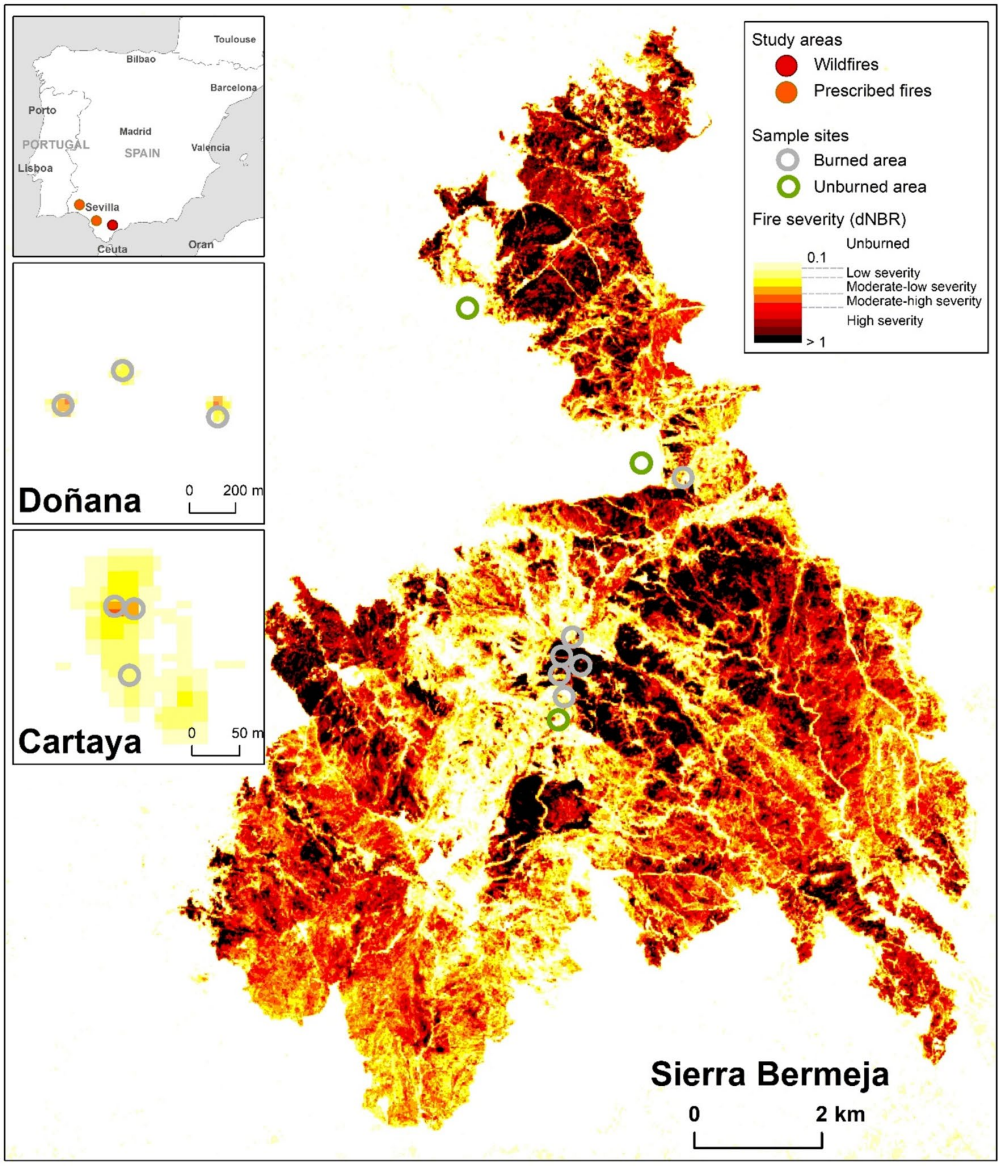
Map representing the different levels of soil burn severity (SBS, dNBR) observed in the Sierra Bermeja wildfire. Coloured circles represent the sampling locations characterised by high and low fire severit**y**. In the upper left corner, map of the Iberian Peninsula, with the 3 study areas represented. Below, maps of the prescribed burning in the Doñana and Cartaya sites, with sampling points in the different levels of SBS.

The mechanisms underlying P limitation in post-fire scenarios have received much less attention than those involving soil organic matter (SOM) or N^17,36^. Soil P dynamics are complex due to the different factors involved (SOM, soil pH, clay minerals, texture, etc.) and the uncertainty regarding the chemical nature of organic P forms^37^. Although P dynamics and availability may be crucial during post-fire restoration, few studies have investigated the short-term impacts of fire on P dynamics^28,38^. To our understanding, no previous studies have investigated the different P forms in different post-fire scenarios. Considering the extent of soil P limitation worldwide, its importance for agricultural and forest productivity, and the essential P role in post-fire soil fertility, we hypothesize that (i) different levels of SBS will induce changes in the extractable P forms, especially the most labile in prescribed burning and wildfires, and (ii) SBS will determine short-term P dynamics in both the organic and mineral soil layers in the southern Iberian Peninsula (Fig. 1). Therefore, our main objective was to assess the quantitative and qualitative effects of both PB and wildfires on soil P forms and dynamics over time. More specifically, we assessed (i) the effect of different SBS on soil parameters of PB and wildfires, (ii) the effect of different SBS on soil P of PB and wildfires and (iii) the dynamics of the different soil P forms (organic, inorganic) before (pre-fire), immediately (post-fire) and 1 year after burning by using ^31^P-NMR spectroscopy.

## Results

### Effects of fire on soil pH, SOC, N and C/N

The pH of the organic soil layer was notably affected by PB in both Doñana and Cartaya and the Bermeja wildfire (Table 1). In the PB, the soil pH increased by 2 units immediately after the fire, returning to pre-fire levels after 1 year. By contrast, the pH in the Bermeja wildfire increased significantly by one and two units for the low and high SBS, respectively, and remained high one year later. In both types of fire (wildfire and PB), the pH of the mineral soil layers (0–2, 2–5 cm) was not affected.

**Table 1:** Changes in soil pH, SOC, N and C:N (mean ± SD) in the organic layer and the mineral layers (0–2 cm, 2–5 cm) of the prescribed fires of Cartaya and Doñana and Bermeja wildfires (low and high).

	Soil layer	Doñana			Cartaya			Bermeja LOW			Bermeja HIGH		
		Prefire	Postfire	1 year later	Prefire	Postfire	1 year later	Unburned	Postfire	1 year later	Unburned	Postfire	1 year later
pH	Organic	**4.3 ± 0.2b**	**6.6 ± 0.3a**	**5.0 ± 0.3b**	**4.2 ± 0.1b**	**6.6 ± 2.0a**	**4.6 ± 0.1b**	**5.9 ± 0.5b**	**7.0 ± 0.3a**	**7.3 ± 0.3a**	**5.9 ± 0.5b**	**8.0 ± 0.3a**	**7.8 ± 0.04a**
	0–2 cm	4.8 ± 0.5	5.5 ± 0.6	5.2 ± 0.5	4.1 ± 0.4	4.8 ± 0.1	4.7 ± 0.5	6.4 ± 0.7	6.3 ± 0.3	7.1 ± 0.4	6.4 ± 0.7	6.6 ± 0.6	7.1 ± 0.2
	2–5 cm	5.2 ± 0.5	5.5 ± 0.4	5.0 ± 0.3	4.5 ± 0.1	4.6 ± 0.4	4.6 ± 0.3	6.5 ± 0.4	6.1 ± 0.3	6.8 ± 0.04	6.5 ± 0.4	6.2 ± 0.2	7.1 ± 0.1
SOC (%)	Organic	**34.9 ± 6.3a**	**6.3 ± 1.8c**	**22.7 ± 2.9b**	**43.3 ± 2.2a**	**11.7 ± 0.9b**	**18.3 ± 2.8b**	25.3 ± 9.5	21.3 ± 11.1	15.9 ± 6.6	**25.3 ± 9.5a**	**3.94 ± 0.8b**	**15.1 ± 3.5ab**
	0–2 cm	2.4 ± 1.9	1.04 ± 0.3	2.3 ± 1.8	**19 ± 11.5a**	**3.5 ± 1.0b**	**3.7 ± 2.0ab**	9.36 ± 7.2	7.3 ± 5.1	6.35 ± 2.6	9.4 ± 7.2	5.26 ± 2.2	4.9 ± 1.2
	2–5 cm	0.8 ± 0.4	0.8 ± 0.1	1.1 ± 0.3	3.4 ± 1.2	2.2 ± 0.4	5.3 ± 3.7	3.66 ± 0.9	5.3 ± 4.5	5.96 ± 0.5	3.7 ± 0.9	3.26 ± 1.7	5.96 ± 2.2
Total N (%)	Organic	**0.8 ± 0.2a**	**0.25 ± 0.1b**	**0.7 ± 0.04ab**	**1.14 ± 0.1a**	**0.8 ± 0.3ab**	**0.5 ± 0.05b**	0.83 ± 0.4	0.9 ± 0.3	0.82 ± 0.4	**0.83 ± 0.4a**	**0.21 ± 0.1b**	**0.45 ± 0.1ab**
	0–2 cm	0.14 ± 0.1	0.06 ± 0.02	0.13 ± 0.1	**0.59 ± 0.3a**	**0.17 ± 0.1b**	**0.15 ± 0.04ab**	0.40 ± 0.3	0.32 ± 0.2	0.3 ± 0.01	0.4 ± 0.3	0.22 ± 0.1	0.16 ± 0.01
	2–5 cm	0.05 ± 0.02	0.07 ± 0.02	0.08 ± 0.002	0.14 ± 0.04	0.10 ± 0.03	0.21 ± 0.13	0.20 ± 0.05	0.24 ± 0.2	0.23 ± 0.1	0.2 ± 0.05	0.12 ± 0.03	0.11 ± 0.02
C/N	Organic	**44.01 ± 3.9a**	**25.5 ± 4.1b**	**33.3 ± 4.4ab**	**38.4 ± 4.9a**	**20.7 ± 8.2b**	**37.2 ± 1.9a**	**32.8 ± 7.7a**	**22.8 ± 4.3ab**	**19.8 ± 1.5b**	**32.8 ± 7.7a**	**20.8 ± 5.02b**	**33.1 ± 1.8a**
	0–2 cm	18.9 ± 12.7	16.98 ± 2.8	16.52 ± 4.9	30.1 ± 6.45	20.96 ± 1.2	24.6 ± 6.3	22.01 ± 3.5	22.6 ± 2.04	20.6 ± 3.9	**22.01 ± 3.5b**	**22.6 ± 2.2ab**	**30.8 ± 6.5a**
	2–5 cm	21.7 ± 12.8	12.40 ± 3.9	14.69 ± 3.95	24.45 ± 4.2	24.0 ± 3.93	23.4 ± 3.9	18.4 ± 0.7	20.8 ± 2.5	26.6 ± 8.3	18.4 ± 0.7	26.9 ± 9.2	34.4 ± 10.1

Results highlighted in bold indicate differences (in the mean values) within the same site. Different letters indicate significant differences within the same soil layer at *P* ≤ 0.05 between different times using Dunnet T3 as the post-hoc test.

The opposite trend was observed in the C and N concentrations. In all cases, PB and wildfires greatly decreased C and N levels in the organic layer (Table 1). Immediately after fire, the C and N concentrations in the Doñana PB were significantly reduced by 82% and 69% respectively, while in Cartaya the reduction was 54% and significant for both C and N concentrations. In the Bermeja wildfire, the C and N concentrations were not significantly reduced in the low SBS sites (Table 1) while significant decreases in C (74%) and N concentration (62%) and consequently reduced C:N ratio (*P* = *0.016*) were observed in the high SBS sites. The C and N concentrations in the mineral soil only decreased in Cartaya, with significant reductions of 81% and 71%, respectively for the C and N concentrations in the 0–2 cm layer.

After one year, the C concentration in the organic layer partly recovered to pre-fire levels. Reductions of 35%, 49%, 46% and 40% were observed for Doñana, Cartaya and the Bermeja low and high SBS sites. The N concentration 1 year later almost reached pre-fire values, and the reduction was very low, being notable only below 15%, 48%, 1% and 46% the pre-fire levels, and with no significant differences, except in Cartaya (*P* < 0.001). The C:N ratio in both cases decreased rapidly after the fire, followed by recovery after one year. The outputs of the three GLMs models developed are shown in Table S1. Model 1 (*site* ~ *time* ~ *layer*) provided the best fit to the data and was better for explaining variance than model 2 (*fire nature* ~ *time* ~ *layer*) and model 3 (*SBS* ~ *time* ~ *layer*).

### Post-fire changes in soil extractable P and different P forms

#### Soil extractable P

**Fig. 2:**
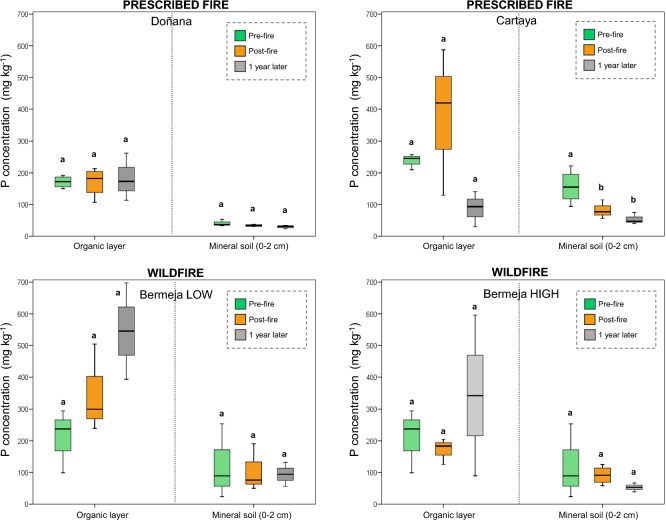
Phosphorus (P) concentration (mg kg^−1^) in the Doñana and Cartaya prescribed fires and the Bermeja wildfires (low and high severity). Different letters indicate significant differences at *P* ≤ 0.05. The dotted vertical line separates the results of the organic layer (left) and the mineral soil (right).

**Fig. 3:**
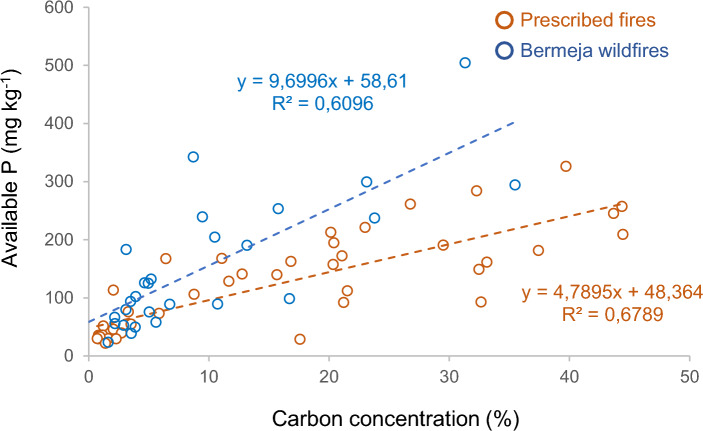
Linear relationship between available P (mg kg^−1^) and C concentration (%) in the prescribed fires of Doñana and Cartaya (orange circles) and Bermeja wildfires (blue circles), for all soil samples collected at the pre-fire, post-fire and 1 year later.

The concentration of extractable P in the organic layers before PB was lower in Doñana than in Cartaya (Fig. 2). Immediately after the fire, the P concentration increased in both sites, and the same was observed for the Bermeja low SBS wildfire, although the differences were not significant. However, the P concentration decreased in the organic layer of Bermeja soils affected by high SBS. In the mineral soils (0–2 cm) the P concentration only decreased significantly, relative to pre-fire levels in Cartaya, by 54% (*P* = 0.007) immediately after the fire and by 70% (*P* = 0.007) one year later (Fig. 2). In addition, in both prescribed and wildfires, the P (mg kg^−1^) and C (%) concentrations were positively related (Fig. 3).

**Fig. 4:**
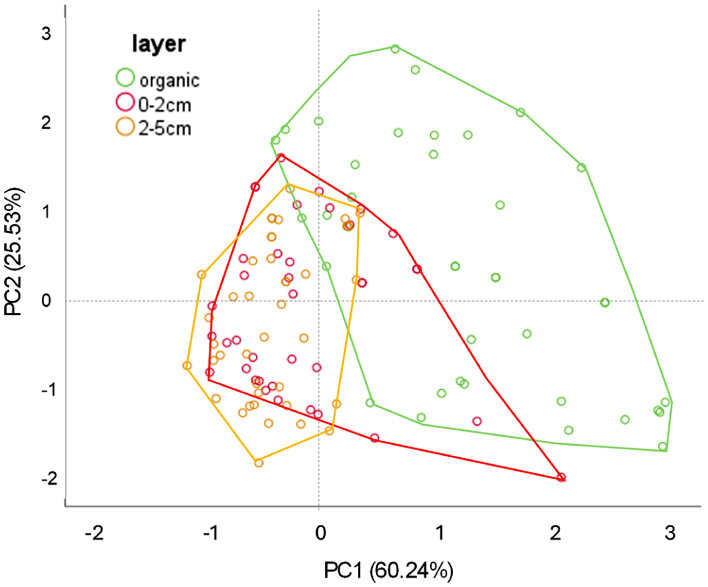
PCA plot representing the bidimensional distribution of soil data (pH, C, N, P, and C/N) according to the different soil layers. PC1 accounted for 60.24% of the variance while PC2 accounted for 25.53% of the variance. Green polygon represents organic layer data, red polygon represents data from surface mineral layer (0–2 cm) and orange polygon data from 2 to 5 cm layer.

The PCA analysis provided four main principal components explaining 99.57% of the total variance (Fig. 4). PC1 and PC2 accounted for 85.77% of the total variance. The first component was associated with C, N and P (positive loadings > 0.79), while PC2 was mainly influenced by pH (loading = − 0.87). The PCA biplot also indicated that soil layers should be explored separately. The organic, the 0–2 cm and the 2–5 cm mineral layers are spatially separated in Fig. 4, with a large number of data points overlapping, which suggests that soil layers share some information as layers are not isolated compartments.

#### Effects of fire on soil P forms in the organic layer

**Fig. 5:**
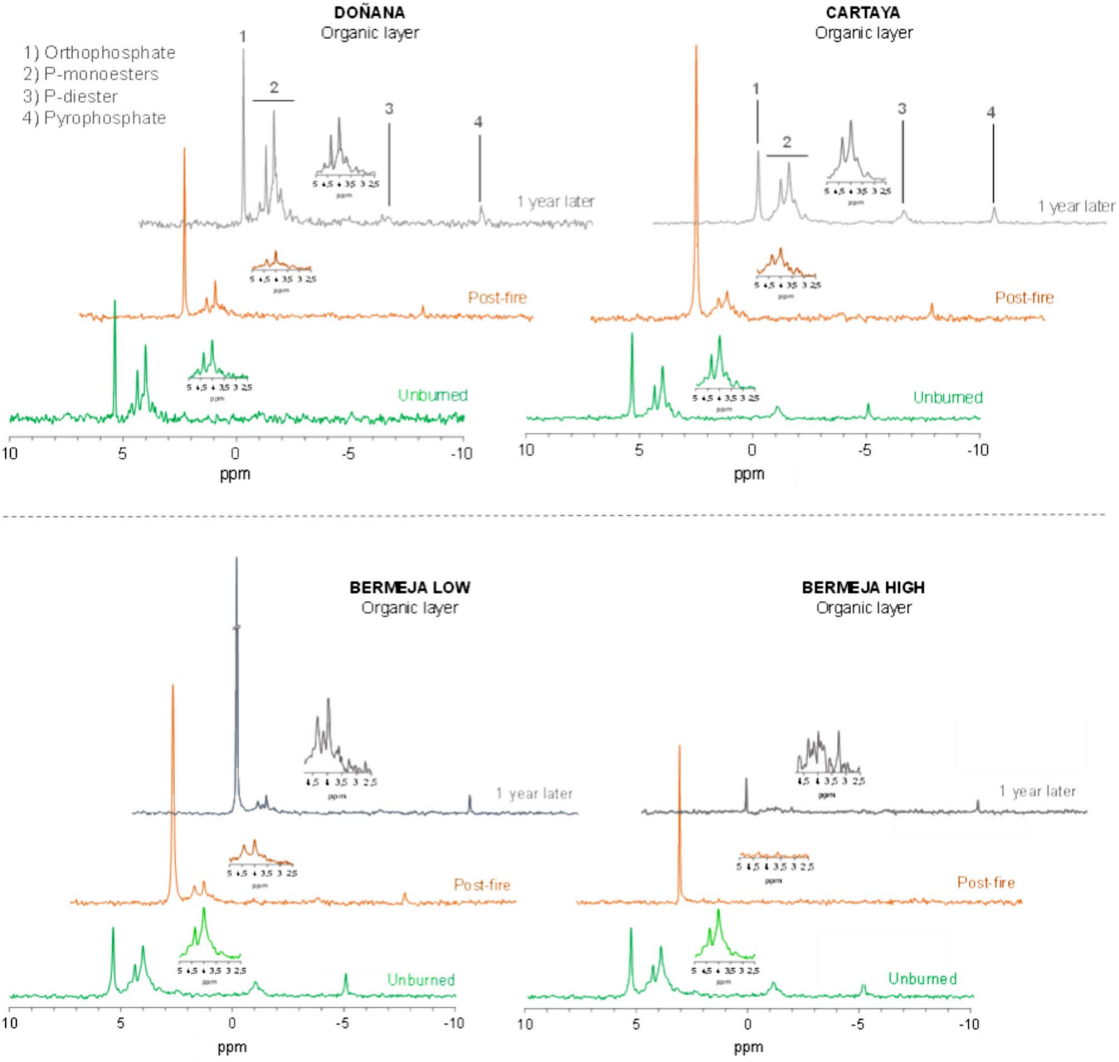
Different ^31^P-NMR spectra of the organic soil layers of Doñana (upper left), Cartaya (upper right) Bermeja low (lower left), and Bermeja high (lower right) in the different time periods (unburnt, post-fire and 1 year later). *The peak corresponding to orthophosphate in the *1 year later* series in Bermeja low is truncated for representation purposes.

**Fig. 6:**
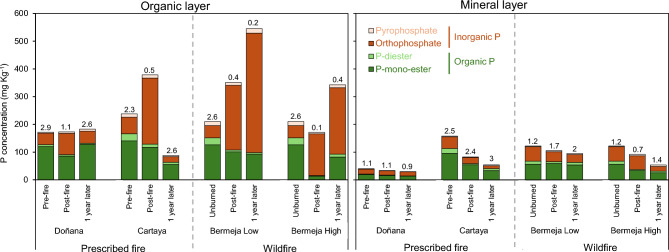
Concentrations of the different forms of P in treatments (pre-fire, post fire, 1 year later) based on the areas obtained in the ^31^P NMR spectra. Stacked bars represent P fractionation in the organic layer (left), and the mineral layer (right). Organic P forms (P-diester, P monoesters) are shown in green, while inorganic P forms (orthophosphate, pyrophosphate) are shown in brown. The number above each bar indicates the *Po:Pi* ratio. For a detailed consideration of each fraction consult Supplementary Table 2.

The ^31^P NMR spectra showed noticeable changes in the relative distribution of P forms in the organic soil horizons (Fig. 5). The pre-fire P forms in the soil organic layers of Doñana and Cartaya were mainly P-monoesters (70–59%, respectively), followed by orthophosphate (24–25%), P-diester and pyrophosphate (Fig. 6). Within the P-monoesters, β-glycerophosphate was the dominant compound (21 and 28%, respectively), followed by myo-inositol in Doñana (18%) and phosphatidic acid (13 and 12%) (Table S2). Considering the most labile organic P forms (phosphatidic acid, β-glycerophosphate and diester), the sum of these proportions accounted for 38 and 51% of available P, respectively. On the other hand, the slow turnover of P_o_ forms (*myo-inositol* and *scyllo-inositol*) represented 20 and 11% of available P in Doñana and Cartaya, respectively. The distribution of the different P forms in the unburnt organic layer in the Bermeja soil was similar (Fig. 6). We did not detect either phosphonates (recalcitrant P compounds with a C-P bond, usually appearing at 20 ppm) or polyphosphates (appearing at − 20 ppm). However, orthophosphate (5 ppm) and monoesters (2–3 ppm) were easily recognized in both the organic (Fig. 5) and mineral soil layers (Fig. S1).

In the organic horizon, the relative distribution of the different P forms changed greatly after PB (Fig. 5). Notable increases in orthophosphate P were observed immediately after the PB, with a 2.5-fold increase in the Cartaya site, and almost doubling its concentration (× 1.9) in Doñana. As expected, the heating led to a significant reduction in P_o_ forms (both diester and monoester P) in both Doñana (− 29.7%) and Cartaya (− 52%) (Fig. 6). The pyrophosphate concentrations did not follow any clear trend, increasing slightly in Doñana (by 2 to 3%) but decreasing in Cartaya (by 3 to 5%). On the other hand, the *P*_*o*_*:P*_*i*_ ratio (proportion of organic: inorganic P) decreased consistently with increasing SBS, being 1.1 in Doñana, 0.5 in Cartaya, 0.4 in Bermeja-low and 0.1 in Bermeja-high (Fig. 6; Table S2). Interestingly, the *P*_*o*_*:P*_*i*_ ratio and also other P forms gradually recovered after one year. Thus, 1 year after the PB, the orthophosphate decreased to pre-fire values and monoesters returned to the initial levels in Cartaya (63 to 59%) and Doñana (70% in both cases). Accordingly, the organic P fraction gradually recovered, and the *P*_*o*_*:P*_*i*,_ ratio returned to pre-fire values.

Wildfire also led to substantial changes in the organic layer of Bermeja soils. Immediately after fire, the amount of orthophosphate increased substantially: a threefold increase in the case of Bermeja low SBS (21 to 67%), and up to 4 times in Bermeja high SBS soils (21 to 88%) (Fig. 6). By contrast, the values of all organic forms of P underwent a significant decline; monoesters were reduced in soils affected by low and high SBS (52 and 87%, respectively), while P-diesters also decreased in low and high SBS (83 and 92%, respectively). Pyrophosphate was similarly reduced by 57% by both levels of SBS. Different levels of burn severity completely altered the *P*_*o*_*:P*_*i*_ ratio. Large increases in orthophosphate significantly reduced the proportion of the organic P fraction, from 2.6 to 0.4 for low SBS and 0.1 for high SBS. After one year, the levels of organic P in Bermeja soils affected by low or high SBS did not return to the levels observed in unburnt soils. The inorganic P forms in both low and high SBS did not vary substantially between the post-fire and 1 year later periods, with orthophosphate predominating (67–80%). The presence of organic forms, such as monoester, was very low (8–29%).

#### Fire effects on soil P forms in the mineral layer (0–2 cm)

In all soils studied, the proportions of the different P forms in the mineral layer before the fire showed a dominance of P-monoesters over orthophosphate (Fig. S1). The dominance was higher in Cartaya (61% over 27%) than in Doñana (49% over 44%). Similar proportions remained (Fig. 6) immediately after the fire, but 1 year later dominance of P-monoester was only observed in Cartaya, in which orthophosphate decreased to 19%. The most significant change was observed in the distribution of the different P-monoesters, particularly in Doñana (Table S2). Immediately and one year after the PB, the *P*_*o*_*:P*_*i*_ ratio in Doñana was slightly affected, but the positive values were maintained (1.1 and 0.9). In Cartaya, the ratio increased slightly, with similar positive values being maintained (2.5 and 3.0) (Fig. 6).

The results for the mineral soils affected by Bermeja wildfires were similar to those of the PB (Fig. 6). The dominance of P-monoesters over orthophosphate appeared in unburnt Bermeja soils (46% over 43%) and remained after the low severity wildfire (56% over 34%); however, the opposite trend was observed in the high SBS wildfire (37% over 54%) (Fig. 6, Table S2). Nonetheless, one year after the wildfire, the dominance of monoester over orthophosphate was again observed in both the Bermeja-low (57% and 30%) and Bermeja-high (48% and 32%) sites. This was equally reflected in the increase of the *P*_*o*_*:P*_*i*_ ratios, from 1.7 to 2 in Bermeja low and from 0.7 to 1.4 in Bermeja high. The predominance of orthophosphate was still observed in the Bermeja low site 1 year after fire (Fig. 6, Table S2). Neither phosphonates nor polyphosphates were detected.

## Discussion

### Effects of fire on soil pH, C and N

The study findings showed that changes in the soil pH and C and N concentrations strongly depend on fire severity and less so on the type of fire (prescribed/wildfire). Increases in SBS were associated with steady increases in pH and decreases in C and N concentrations^3,12,39^. Combustion of SOM and deposition of ashes containing alkaline compounds increased the pH by 2–3 units in the organic fraction, while the C concentration in the organic and mineral soil fractions decreased almost linearly with fire severity, as previously observed^12,40^. As a consequence, nutrients stored in the SOM are rapidly released after combustion, thus facilitating, if the physical soil properties allow, the initial recovery of vegetation in areas affected by low SBS^41^. Nevertheless, plant growth can be constrained, with negative long-term consequences due to consumption of soil nutrient stocks (C, N and P)^42^ or reduced N availability due to differences in N and P losses in the short-term^43^.

One year after both PB events, the pH levels recovered, but C concentrations remained below pre-fire levels in all the areas. For both of the wildfires, the minimum recovery observed one year after the wildfire may endure over time, as previously reported in other Mediterranean areas affected by low soil burn severity^8^. The high pH values observed in the Bermeja sites after one year may also be affected by the alkaline nature of the parent material^44^. The higher amount and availability of fuel in Cartaya soils (SOM before PBs was almost 80% of the total mass) may have triggered a stronger fuel consumption during the fire and a slow recovery of C and N concentrations, as previously observed after PB^4,45^. In the case of N recovery, a similar trend to that of C was observed, with the Doñana soils being similar to those in the Bermeja site affected by low SBS and Cartaya soils similar to those in the Bermeja site affected by high SBS.

### Pre-fire P soil forms

The available P concentration in the organic soil layer in the study sites is comparable to values reported in other studies on Mediterranean soils^28^. The concentrations of extractable P in the mineral soil (36–141–190 mg kg^−1^) were similar to those reported in other studies in similar burnt areas (10 and 155 mg kg^−1^, respectively for^42^ and^46^). In the Bermeja and Cartaya soils, the P levels were relatively high before the fire events^28^. Prior to the fires, the soils contained a high proportion of organic P (60–70%), as observed in previous studies across the Iberian Peninsula^27,28,38,47^. Most of the P is probably associated with the soil organic fraction^38,48^, as shown by the strong linear correlations between P and C concentrations in our data, including pre- post- and 1 year after fire and organic and mineral layers (Fig. 3).

Both the litter and the mineral soil in the three sites contained organic P forms with higher proportions of P-monoesters than diester^24,28,37^, and they were mainly represented by monoesters closely associated with the SOM^24,37^. P mineralization is controlled by a combination of biological and biochemical factors^29^ and P concentrations, and P availability and leaching rates are considered generally low in unmanaged forest^31^. However, fire can influence P availability in a similar way to natural mineral weathering^48^. The high monoester:diester ratios in unburnt organic layers in the Bermeja and Cartaya (6:1) and Doñana (14:1) soils are consistent with previous findings^26,27,38,49^. In undisturbed soils, monoesters accumulate preferentially, as these P forms are susceptible to sorption due to high charges^50^ or the formation of stable complexes resistant against microbial activity^29^. Monoesters will only begin to be consumed when P is limited. By contrast, due to its intrinsic chemical nature (only one ionizable proton per phosphate), P diester is considered more labile and its adsorption capacity is therefore reduced, and this form is generally present in soils at lower concentrations than monoesters.

### Post-fire distribution of soil P forms: fire nature or fire severity?

In the present study, we observed that post-fire SOM consumption consistently led to an imbalance in the organic:inorganic P ratio in the organic layer, by reducing organic P while increasing the inorganic P forms, as previously observed^27,28^. Although the SOM-P dynamics may be similar, organic P is more vulnerable to thermal mineralization than SOM^51,52^. Loss of P through volatilization, which has been reported to occur at temperatures between 200 °C^28^ and 800 °C^53^, probably occurred in the high SBS sites, at least in the organic soil layer. Indeed, these authors showed that even low temperatures (± 70 °C) can lead to a significant reduction in the concentration of the most labile P_o_ fraction, a reduction of around 30–50% similar to that observed in Doñana and Cartaya, respectively. In addition, the strong post-fire alkalinization, bringing the pH close to optimum values for P solubilization (6–6.5), favours soil P depletion via solubilization and mobilization with run-off and eroded sediments^54^, which can be detrimental to the maintenance of long-term P supply in the system^28^. Beyond a certain pH threshold, P availability may also be reduced due to physical trapping phenomena (occlusion) in carbonates^11,28^.

In the present study, soils affected by low SBS, as in the Doñana site, produced a short-term orthophosphate peak in the organic layer, possibly due to the mineralization of monoesters and diester^38^. In soils affected by higher SBS, as in the Cartaya and Bermeja sites, the clear dominance of orthophosphate and the disappearance of the remaining P forms was consistent with previous findings in sites affected by high SBS^11^. Along with the orthophosphate dominance, the clear trend of diminishing P-diester with increasing fire severity was another notable effect in our study and elsewhere^27,28^. While roughly 85% of these more labile forms were lost in the Bermeja-Low and Cartaya sites, the P loss was much smaller in Doñana. In both cases, the *P*_*o*_*:P*_*i*_ ratios were almost totally inverted after the fire. Our findings showed that PB acts like medium–low severity fire^3^, affecting the different P forms in the following order of SBS: Bermeja-High (4.8) > Bermeja-Low (2.7) ~ Cartaya (3.0) > Doñana (2.5). As a consequence, the most important factor explaining the changes in soil P dynamics was fire severity, rather than the type of fire.

### Changes in soil P and P forms one year after fire

To date, the time taken to recover the different P forms after prescribed or wildfires has scarcely been investigated. After PB, we observed slight decreases in mono- and diesters in organic horizons, while the strong initial increase in orthophosphate returned to pre-fire levels after one year. Conducting PB is intended to ensure low impact on the forest ecosystem, and this type of fire is generally characterized by low SBS, with rapid recovery of soil and vegetation during the first to third years post fire^5^. However, the present findings indicate that despite low aboveground intensity, low SBS or low peak temperatures, PB may sometimes have strong effects, possibly due to the longer fire residence time, as in Cartaya, with a mean residence time of 22 min of T > 100 °C^11,38^. By comparison, fire spread in the Sierra Bermeja wildfire was estimated to be much faster, around 25–30 m min^−1^. The risk of post-fire soil erosion that may arise in steep, difficult-to-access hillslopes, even in areas affected by low SBS, has already been pointed out^10^. We observed that the accumulation of organic debris may increase fire intensity and lead to higher-than-expected SBS and fire residence times, as probably occurred in the Cartaya PB, thus delaying the recovery of pre-fire values of pH, C, N or available P beyond the first post-fire year.

The strong initial post-fire reduction in P diester, mainly the rapid mineralization of P forms from nucleic and phospholipids microbial constituents, reflects the decay in microbial biomass^25,55^, an effect comparable to the reduction in microbial activity and diversity observed in intensively managed agricultural soils^56^. Consequently, the changes in P-diesters will depend on the recovery of soil microorganisms in the post-fire scenario. One year after fire, the proportion of P-diester increased in a different way, indicating an improvement in soil health, especially in the organic layer, in which the P_o_ forms are closely associated with SOM^29^, suggesting the recovery of microorganisms that can immobilize P. The strong reduction in SOM and the increasing recalcitrance of C forms, mainly at high SBS, also represents an initial barrier to microbial recovery, leading to slower recovery of P-diester.

On the other hand, the peridotite-derived mineral soils of Bermeja, with a high pH of around 6.5, favoured the complexation and permanence of orthophosphate in both the low and high SBS soils 1 year after the fire. Ash accumulation, especially with limited erosion conditions due to a carpet of needles^40^, can also explain the high orthophosphate concentration observed in low SBS after 1 year. However, although the soil was rapidly covered by a thin layer of pine needles, the acidic pH (4.1) of the mineral soil in Cartaya probably limited orthophosphate complexation. The ash and charred biomass inputs from understorey, dead downed wood and branches from the tree canopy represent other sources of Pi over time^38^. In this respect, the biomass inputs in the Bermeja and Cartaya sites (pine-dominated vegetation) must have been similar, but different from those in Doñana (shrub-dominated vegetation). However, this was not the case in the Doñana and Cartaya PBs, in which a similar trend in recovery of the pre-fire P_o_ levels was observed. The differences in P dynamics between PBs and wildfires may have masked an effect of a different pH, as both Bermeja sites (affected by low and high SBS) had very high levels of ortophosphate and the pH was the same. In this respect, our findings do not allow us to separate unequivocally the effects of wildfires and PBs: while monoesters and di-esters did not recover one year after the wildfires, the orthophosphate and total P levels were maintained, and even increased substantially. The nature of soils may have interfered with P dynamics, favouring the conservation of some P forms (orthophosphate) and the loss of others (mono- and di-esters). Further research is essential to unravel post-fire P dynamics and the recovery of the different P forms in other soil types.

### Implications for forest management and restoration

The long-term supply of P in forest ecosystems is closely associated with the soil OM. Organically-complexed P forms ensure the maintenance of soil P pools, which are essential for soil productivity in the long-term^57^. A greater diversity of P forms has been found to be positively related to a greater provision of ecosystem services (such as nutrient retention, soil C stability and soil biogeochemical functions) across different types of land use^58^.

The selection of areas for conducting post-fire soil interventions after wildfires should consider fire severity criteria, such as the SBS index^12^. We have found that SBS strongly affects the relative distribution of P forms in burnt soils, as lower levels of P diesters were still observed in the organic fraction of the Doñana and Bermeja-low soils after one year. The subsequent changes in P forms will be strongly conditioned by the soil and probably also by climate factors. Thus, environmental constraints such as the sandy parent material and the limited access to plant propagules in Doñana or the ultrabasic parent material of the Bermeja site probably limited vegetation recovery during the year after fire. In areas of high SBS, intervention is often required, and soil conservation efforts must be implemented to prevent degradation and promote revegetation^40^. Thus, if erosion occurs, the richest fraction of soils will be lost, and the bulk of soil nutrients will be exported off-site. This is particularly important in regions with a high risk of post-fire erosion, as some of those included in our study^42,59^.

Immediately after fire, the greater amount of P in the soil solution -mainly the mobile inorganic orthophosphate- is readily available to plants. However, the increase in the proportion of readily available P forms may facilitate the process of P ageing^37^ and after wildfire the soil system faces the potential reduction of P availability in the long term^31^. One year after PB that led to low to moderate SBS, we observed that the organic P recovered (particularly monoesters), which (in terms of P availability) supports the feasibility of using PB as a forest management tool. Nevertheless, it must be taken into account that, despite the lower associated risk, the cumulative effect of carrying out repeated PB can have unforeseen effects^14^.

## Conclusions

The study identified, for the first time, post-fire changes in P forms and dynamics in the organic and mineral surface soil layers of Mediterranean woodlands affected by prescribed burning and wildfires during the first year of recovery. Important parameters for soil conservation (pH, C, N, C:N) showed some symptoms of recovery after PB or wildfires, and even returned to pre-fire values, as in the mineral soil layers, but to a lesser extent in the organic soil layer.

Although the post-fire effects were similar, PB and wildfires had notably different effects associated with SBS, maximum soil temperature reached by the fire and mean fire residence time. In PBs, the *P*_*o*_*:P*_*i*_ ratios recovered after 1 year, even increasing in both the organic and the mineral soil fractions in all soil horizons and sites, except in the mineral soil in Doñana. By contrast, the wildfires had a severe impact on the P organic fraction. High SBS and to a lesser extent low SBS led to the complete P mineralization in the organic horizon. One year after the PB, organic P had not recovered, indicating a clear difference related to fire severity. Rather than focusing on the prescribed burning/wildfire dichotomy, our findings suggest that SBS is the key factor explaining the changes in soil P dynamics.

Prescribed burning has a strong potential to reduce fire risk, and trade-offs have been addressed in recent years. Our findings indicate that after low SBS reached in either PB or wildfire, different P forms recovered to pre-fire levels during the first year after fire.

## Materials

### Prescribed burning (PB). Doñana and Cartaya sites

The Doñana PB (37°01′17.3″N 6°28′41.9″W) was conducted in a sparse shrubland within the Doñana National Park, south of Seville (Fig. 1). The climate in the region is classified as hot summer Mediterranean (Csa, Koppen classification), with an annual average rainfall of 550 mm and annual average temperature of 17 °C. The sclerophyllous vegetation is composed of a gradient ranging from the driest “monte blanco” dominated by germinators that are strongly adapted to fire (*Cistus libanotis* L., *Halimium calycinum* (L.) K.Koch, *H. halimifolium* (L.) Willk.**,*** Salvia Rosmarinus* Spenn., *Lavandula stoechas* Lam. and *Thymus mastichina* L. and resprouters such as *Stauracanthus genistoides* (Brot.) Samp*.*, to the intermediate “monte intermedio” (composed of *H. halimifolium* and *Ulex australis* Clemente) and the wettest “monte negro” (including resprouters such as *Erica scoparia* L. and some germinators such as *Calluna vulgaris* (L.) Hull)^60^. The soils ranged from Albic Arenosols to Dystric Gleysols, saturated by water during winter (IUSS Working Group WRB, 2015). The main substrate is composed of detritic, acid unconsolidated Plio-Quaternary continental deposits from the Neogene, covered by Quaternary fluvio-marine and aeolian materials^60^.

The Cartaya PB (37°21′59.5″N 7°13′30.2″W) was carried out in the Cartaya pine forestland, west of Seville (Fig. 1). The climate in the region is classified as hot summer Mediterranean (Csa, Koppen classification), with an average temperature of 18.5 °C and average annual rainfall of 500 mm. The forest is dominated by *Pinus pinea* L. stands with understory vegetation mainly formed by a fire-adapted mixture of Mediterranean shrubs, dominated by *Cistus ladanifer* L.*, C. monspeliensis* L.*, C. crispus* L. and *Genista sphaerocarpa* (L.) Boiss*..* The soils are Umbric Inceptisols or Umbrisols (IUSS Working Group WRB, 2015), developed from acidic quaternary sand deposits, and with accumulation of large amounts of organic matter at the mineral soil surface.

#### Prescribed burning

The main objective of the prescribed fires was to reduce fuel accumulation for different reasons. While the Doñana PB was intended to promote plant recruitment and boost recovery of the Iberian Lynx, the objective in Cartaya was mainly to reduce fire hazard in the pine stand. The Doñana PBs affected a flat area of 3 ha, and it was carried out on 28–29 October (2020). The Cartaya PB affected an area of 3–4 ha and was carried out on 5 November, 2019. At the time of the PB the weather conditions for each of the Cartaya-Doñana sites were suitable for initiating medium–low intensity fires (biomass moisture 89–136%, air temperature 22–24 °C, wind speed 1.7–2.2 m s^−1^, respectively). The prescribed burning was carried out early in the morning by setting narrow strips of fire (2–5 m) upwind so that the head fire could not reach high energy level and thus to ensure a low intensity fire. Prior to burning, vegetation was cleared in a 5-m strip around the perimeter of the target area for safety purposes. In the Doñana PB, the soil burn severity (SBS)^12^ ranged from 0 (unburnt) to 3 (all forest floor consumed and some ashes), with an average SBS of 2.5 (± 0.6). In most sites in Cartaya, SBS levels were between 1 (soil organic layer lightly charred, mineral soil not affected) and 4 (thick layer of grey ashes and mineral soil structure affected), with an average SBS of 3.0 (± 1.4). SBS has been used in a wide variety of ecosystems, useful to estimate fire severity in boreal forests, Atlantic and Mediterranean climates^12^. Thermocouples (type K 1 mm diameter) were installed at four positions in each subplot: at the litter surface, at the mineral soil surface, at 2 cm of soil depth and 5 cm of soil depth (Lascar, Easylog, Wiltshire, UK). Thermal regime during PBs showed a mean maximum peak temperature at the litter surface of 727 °C for Doñana, and 566 °C for Cartaya. However, mean residence times (at Tº > 100 °C) were 3.4 min at Doñana and 22.1 min at Cartaya.

### Wildfires. The Bermeja-low and Bermeja-high sites.

Between 8 and 14 September (2021), the Sierra Bermeja mountain range (36°30′47.7″N, 5°11′08.1″W) was affected by a large wildfire **(**Fig. 1**)**, one of the most intense fire events recorded in the region to date. The fire lasted for 1 week and consumed almost 9000 ha of pine forest. Three unburnt hillslopes were selected, along with three hillslopes affected by low SBS (range 2–3; average 2.7 (± 0.6), denoted Bermeja-low sites, and three hillslopes affected by high SBS (range 4–5; average 4.8 (± 0.5), denoted Bermeja-high sites, all of which were very steep (30º), west-facing and about 1000 m.a.s.l.

The climate in the study area is classified as Mediterranean semi-oceanic humid-hyperhumid (Csb, Köppen classification), with abundant rainfall and fog and warm, dry summers. Long-term mean annual rainfall and temperature were recorded at the nearest meteorological station (Sierra Bermeja, 1452 m.a.s.l., 3 km south) and averaged 1023 mm and 12.5 °C. The pre-fire tree vegetation consisted of a dominant autochthonous pine forest (*Pinus pinaster* Aiton.) with the presence of spare cork oaks (*Quercus suber* L.). Tree understorey and open areas are occupied by spare shrubland formed by *Pistacia lentiscus* and accompanied by other Mediterranean shrubs (*Erica arborea* L.*, Chamaerops humilis* L.) and annual plants (*Phillyrea angustifolia* L., *Phlomis purpurea* L., *Rubia peregrina* L. and *Daphne gnidium* L.). The Sierra Bermeja soils are developed from peridotites, an alpine intrusion of ultrabasic rocks that are very rich in serpentinite, iron, aluminum and heavy metals; this parent material limits the development of soils and the establishment of vegetation^61^. The soils are lithic and eutric Lithosols of depth less than 30 cm (IUSS Working Group WRB, 2015).

### Experimental design and soil sample collection

In both prescribed and wildfires, fire severity calculated as the differenced Normalized Burn Ratio (dNBR)^62^ was used as a proxy for soil burn severity (SBS). The dNBR was determined by the difference between the pre-fire NBR and the post-fire NBR. The NBR results from the normalized difference of the spectral values of bands 8 and 12 for Sentinel-2 images. To carry out these calculations we used images from 10/24/2019 (Cartaya, pre-fire), 11/16/2019 (Cartaya, post-fire), 10/13/2020 (Doñana, pre-fire), 02/ 12/2020 (Doñana, post-fire), 09/05/2021 (Sierra Bermeja, pre-fire), and 09/18/2021 (Sierra Bermeja, post-fire). Cloud-free images were selected in prescribed burnings and the wildfire of Bermeja, and as close as possible to the analyzed events to reduce the effect of phenological dynamics. Fire severity follows a gradient of equal dNBR intervals, to allow a better perception of its spatial distribution. Even so, we indicate the fire severity classification thresholds previously established^63^, as they resulted from a study more adjusted to our geographic context, with the exception of the last threshold, for which we turn to^62^. All calculations were performed with QGIS Desktop 3.24.1 (https://qgis.org/).

Soil sample collection was as similar as possible in the PB and the wildfires. In the PB (Cartaya, Doñana), the organic layers (litter and FH, or the corresponding charred layer) and the mineral soil (0–2, 2–5 cm) were sampled immediately before burning, 1–2 days after the burning, and 1 year later. In each PB, 3 square plots of 30 × 30 m were delineated in the centre of the area selected for burning (Fig. 1). Each plot included 5 sampling points (located at the four corners and in the center of the square), which were marked with iron reinforcing bars. First, the organic layer was carefully sampled from a square of 25 × 25 cm in each sampling point, and the mineral soil (0–2, 2–5 cm) sampled with metal rings of thickness 2–3 cm. After the fire, the SBS classes were visually estimated^12^ at each sampling point, the ash was carefully collected with a broom in a 25 × 25 cm square, and the mineral soil (0–2, 2–5 cm) was sampled with metal rings.

On the other hand, pre-fire sampling in the case of wildfires is not possible due to the associated dangers. Therefore, soil sampling was carried out in 3 unburnt hillslopes. Another 3 hillslopes characterised by low SBS and 3 characterised by high SBS were sampled and designated Bermeja-low and Bermeja-high sites respectively. Four sampling points along a 30-m transect were visually examined in each hillslope, to estimate the SBS and sampled in the same way for each unburnt-low–high sites. Four sampling points in each PB and wildfire were selected to determine pH, C, N and P concentrations, and ^31^P-NMR spectroscopic analysis of a representative sample of each treatment was carried out.

### Soil analysis

#### pH, soil organic C, total N and extractable P concentrations

The soil pH was determined in a soil–water suspension (ratio 1:2.5), which was first stirred and left to settle for 10 min^64^. Soil samples (0.5 g) were ground to pass through a 0.1 mm mesh screen for determination of organic C and N concentrations in an automated C analyzer (LECO Elemental Analyzer). Soil samples for P analysis were oven dried (65 °C) to constant weight before being milled (0.25 mm) and extracted^65^. Phosphorus concentrations were determined by inductively coupled plasma optical emission spectrometry (ICP-OES).

#### *Phosphorus fractionation by *^*31*^*P NMR spectrometry*

The use of nuclear magnetic resonance spectroscopy (^31^P-NMR) has improved the study of P, enabling qualitative and quantitative estimation of all P forms (ortho-P, mono-P, monoester-P, diester-P, and poly-P) in samples^49^. NMR has been used to assess soil fertility, to investigate the effects of land use change on nutrient cycling and to relate soil P compounds with soil biogeochemical functions^58,66^.

In the present study, the methodology and analysis of soil samples by NMR was as in previous studies^10,28,38^. Phosphorus was extracted by a slightly modified version^63^ of the method described by Cheesman et al.^67^. Briefly, 1 g aliquots of samples of the organic (either litter or remaining charred material) and the mineral fraction (0–2 cm) were extracted with 30 mL of 0.25 M NaOH and 50 mM EDTA for 16 h, and the extracts were then centrifuged (10 min, 6500 rpm). One mL of 50 mg L^−1^ methylene disphosphoric acid (MDPA) solution was added (as an internal standard) to 20 mL of each extract, and the mixture was frozen at − 80 °C and lyophilized. Three-hundred mg of each lyophilized extract was then redissolved in 0.3 mL of deuterium oxide (D_2_O) and 2.7 mL of a solution containing 1.0 M NaOH and 0.1 M EDTA. This solution was placed in a 5 mm NMR tube for analysis. Spectra were acquired at 25 °C in a Varian VNMRS-500-WB NMR spectrometer at a ^31^P frequency of 202.296 MHz. The recovery delay (0.5 s) was set to optimize and reduce the time of experiments, and other conditions were as follows: 90° pulse of 6 μs, acquisition time, 0.2 s, and broadband 1H decoupling. For each sample, 100,000 scans were acquired. The spectra obtained have a line broadening of 2 Hz.

Spectroscopic signals were assigned to the different P compounds^25,68^: orthophosphate (around 5.3 ppm), orthophosphate monoesters (3–5.1 ppm), pyrophosphates (− 5.5 ppm) and orthophosphate diesters (− 2 to 0 ppm). The following monoesters were detected: phosphatidic acid (4.3 ppm), β-glycerophosphate (3.9 ppm), scyllo-inositol (3.2 ppm) and myo-inositol (5, 4.1, 3.8 to 3.4 ppm). The MDPA internal standard appeared at 16.5 ppm. Peak signal areas in the ^31^P NMR spectra were distinguished and further compared by integration. All spectral processing was carried out with MestreNova software, version 8.1.0 (Mestrelab Research Inc., Santiago de Compostela, Spain).

### Statistical analysis

We used generalized linear models (GLM) to determine whether the independent factors site (Doñana, Cartaya, Bermeja-low, Bermeja-high), time (pre-fire/unburnt, post-fire, 1 year after) and layer (organic layer, 0–2 and 2–5 cm mineral layer) affected the response of dependent soil parameters (pH, C, N, C/N and P). Two additional factors were also established on the basis of the experimental design: type of fire (wildfire or PBs) and fire severity, considered high or low SBS. Three GLMs (considering main effects) were constructed, combining the independent factors **model 1** (*site* ~ *time* ~ *layer)*, **model 2** (*fire nature* ~ *time* ~ *layer*) and **model 3** (*SBS* ~ *time* ~ *layer*), with normal distribution and identity as link function selected, as the response variables are considered continuous. The normality of the model residuals and homogeneity of variance were checked with Shapiro–Wilk and Brown-Forsythe tests, respectively. The overall significance of the model was determined by the omnibus test (likelihood ratio χ^2^ test). The proportion of the variation in the response variable explained by the model (D^2^, glm´s R^2^ equivalent) was estimated from the difference between the deviance for the null model and the estimated model, by using the following expression: D^2^ = (D_0_ − D_model_)/D_0_. Dunett’s T3 multiple comparison test was used as a post-hoc test for pairwise comparisons. Relationships between soil characteristics of burnt areas (prescribed burnings and wildfires) were also evaluated using principal component analysis (PCA) to simplify the interpretation of the analysis of soil properties. PCA was based on the standardized soil variable correlation matrix to explore soil nutrient responses in the different soil layers. Varimax rotation was selected, and KMO and sphericity were tested using Bartlett test. All statistical tests were performed using IBM SPSS Statistics version 23.0 (IBM SPSS Inc., Chicago, IL, USA) software for Windows.

## Supplementary Information

Supplementary Figure S1.

Supplementary Tables.

## Data Availability

The authors declare that the datasets generated and/or analysed during the current study are not publicly available because the supporting project is still ongoing, but data can be available from the corresponding author on reasonable request.
