# Supplementary material for: Fire enhances changes in phosphorus (P) dynamics determining potential post-fire soil recovery in Mediterranean woodlands
Source: Sci Rep. 2024 Sep 17;14:21718. doi: 10.1038/s41598-024-72361-8 (PMC11408694; doi:10.1038/s41598-024-72361-8)
Supplement: Supplementary file 1 — Supplementary Figure S1. [file 41598_2024_72361_MOESM1_ESM.docx]

**Figure S1**. Different ^31^P NMR spectra obtained from the mineral (0-2 cm) soil layers of the PBs of Doñana (upper left), Cartaya (upper right), Bermeja low (lower left), and Bermeja high (lower right) in the different time periods (unburned, post-fire and 1 year later). *The peak corresponding to orthophosphate in the *1 year later* series in Bermeja low is truncated only for representation purposes.
