# Supplementary material for: Fire enhances changes in phosphorus (P) dynamics determining potential post-fire soil recovery in Mediterranean woodlands
Source: Sci Rep. 2024 Sep 17;14:21718. doi: 10.1038/s41598-024-72361-8 (PMC11408694; doi:10.1038/s41598-024-72361-8)
Supplement: Supplementary file 2 — Supplementary Tables. [file 41598_2024_72361_MOESM2_ESM.docx]

**Supplementary table S1.** Description of the three generalized linear models (GLMs) output considering the soil parameters (pH, C, N, C/N and P) and the independent factors “Site” (Doñana, Cartaya, Bermeja low, Bermeja high), “Time” (pre-fire, post-fire, 1 year later), “Layer” (organic layer, 0-2 cm, 2-5 cm), “Fire nature” (wildfire, prescribed fire) and “Fire severity” (high and low severity). D=deviance; D^2^ = (D_0_ - D_model_) / D_0_

|  | **pH** |  | **C** |  | **N** |  | **C/N** |  | **P** |  |
| --- | --- | --- | --- | --- | --- | --- | --- | --- | --- | --- |
| **Model 1 (site ~ time ~ layer)** | χ^2^ | Sig. | χ^2^ | Sig. | χ^2^ | Sig. | χ^2^ | Sig. | χ^2^ | Sig. |
| Omnibus test | 131.2_(df=7)_ | *<0.001* | 10016.9_(df=7)_ | *<0.001* | 9.053_(df=7)_ | 0.249 | 3404.24_(df=7)_ | *<0.001* | 748036.8_(df=6)_ | *<0.001* |
| D^2^ |  | 0.663 |  | 0.65 |  | 0.657 |  | 0.35 |  | 0.454 |
| **Test of model effects (Type III)** |  |  |  |  |  |  |  |  |  |  |
| Source | χ^2^ | Sig. | χ^2^ | Sig. | χ^2^ | Sig. | χ^2^ | Sig. | χ^2^ | Sig. |
| (Intercept) | 11021.1_(df=1)_ | *<0.001* | 280.94_(df=1)_ | *<0.001* | 432.69_(df=1)_ | *<0.001* | 1551.02_(df=1)_ | *<0.001* | 165.50_(df=1)_ | *<0.001* |
| Site | 215.67_(df=3)_ | *<0.001* | 11.17 _(df=3)_ | *0.011* | 33.13 _(df=3)_ | *<0.001* | 12.46_(df=3)_ | *0.006* | 24.37_(df=3)_ | *<0.001* |
| Time | 40.69_(df=2)_ | *<0.001* | 33.8 _(df=2)_ | *<0.001* | 17.73 _(df=2)_ | *<0.001* | 19.92_(df=2)_ | *<0.001* | 0.22_(df=2)_ | 0.896 |
| Layer | 6.44_(df=32)_ | *0.040* | 125.40_(df=2)_ | *<0.001* | 146.16_(df=2)_ | *<0.001* | 36.11_(df=2)_ | *<0.001* | 46.84_(df=1)_ | *<0.001* |
|  |  |  |  |  |  |  |  |  |  |  |
| **Model 2 (fire nature ~ time ~ layer)** | χ^2^ | Sig. | χ^2^ | Sig. | χ^2^ | Sig. | χ^2^ | Sig. | χ^2^ | Sig. |
| Omnibus test | 125.81_(df=5)_ | *<0.001* | 9515.6_(df=5)_ | *<0.001* | 13.76_(df=5)_ | 0.152 | 2845.7_(df=5)_ | *<0.001* | 655836.7_(df=4)_ | *<0.001* |
| D^2^ |  | 0. 636 |  | 0.61 |  | 0.586 |  | 0.288 |  | 0.398 |
| **Test of model effects (Type III)** |  |  |  |  |  |  |  |  |  |  |
| Source | χ^2^ | Sig. | χ^2^ | Sig. | χ^2^ | Sig. | χ^2^ | Sig. | χ^2^ | Sig. |
| (Intercept) | 11555.62_(df=1)_ | *<0.001* | 258.72_(df=1)_ | *<0.001* | 339.39_(df=1)_ | *<0.001* | 1417.69_(df=1)_ | *<0.001* | 156.57_(df=1)_ | *<0.001* |
| Fire nature | 205.11_(df=1)_ | *<0.001* | 0.29 _(df=1)_ | 0.58 | 1.31_(df=1)_ | 0.25 | 0.0003_(df=1)_ | 0.98 | 6.55_(df=1)_ | *0.01* |
| Time | 35.12_(df=2)_ | *<0.001* | 30.30 _(df=2)_ | *<0.001* | 16.32_(df=2)_ | *<0.001* | 18.46_(df=2)_ | *<0.001* | 0.15_(df=2)_ | 0.92 |
| Layer | 6.33_(df=2)_ | *0.042* | 117.25_(df=2)_ | *<0.001* | 116.21_(df=2)_ | *<0.001* | 36.31_(df=2)_ | *<0.001* | 42.86_(df=1)_ | *<0.001* |
|  |  |  |  |  |  |  |  |  |  |  |
| **Model 3 (SBS ~ time ~ layer)** | χ^2^ | Sig. | χ^2^ | Sig. | χ^2^ | Sig. | χ^2^ | Sig. | χ^2^ | Sig. |
| Omnibus test | 168.061_(df=5)_ | *<0.001* | 5858.3_(df=5)_ | *<0.001* | 5.725_(df=5)_ | 0.154 | 6467.6_(df=5)_ | *<0.001* | 1074044_(df=4)_ | *<0.001* |
| D^2^ |  | 0.15 |  | 0.62 |  | 0.584 |  | 0.344 |  | 0.372 |
| **Test of model effects (Type III)** |  |  |  |  |  |  |  |  |  |  |
| Source | χ^2^ | Sig. | χ^2^ | Sig. | χ^2^ | Sig. | χ^2^ | Sig. | χ^2^ | Sig. |
| (Intercept) | 3958.03_(df=1)_ | *<0.001* | 256.82_(df=1)_ | *<0.001* | 351.01_(df=1)_ | *<0.001* | 1555.50_(df=1)_ | *<0.001* | 144.88_(df=1)_ | *<0.001* |
| Fire severity | 0.08_(df=1)_ | 0.77 | 0.94_(df=3)_ | 0.33 | 0.53_(df=1)_ | 0.46 | 11.13_(df=1)_ | *<0.001* | 0.03_(df=1)_ | 0.85 |
| Time | 24.48_(df=2)_ | *<0.001* | 30.48_(df=2)_ | *<0.001* | 16.48_(df=2)_ | *<0.001* | 20.02_(df=2)_ | *<0.001* | 0.23_(df=2)_ | 0.89 |
| Layer | 3.92_(df=2)_ | 0.14 | 115.28_(df=2)_ | *<0.001* | 115.27_(df=2)_ | *<0.001* | 36.22_(df=2)_ | *<0.001* | 39.51_(df=1)_ | *<0.001* |

**Supplementary Table S2.** Relative distribution (%) of the different P forms measured by ^31^P NMR in the organic layer and the mineral layers (0-2 cm, 2-5 cm) of the prescribed fires of Cartaya and Doñana and Bermeja wildfires (low and high). **Organic P** is the sum of P-monoesters and P-diester; inorganic P is the sum of orthophosphate and pyrophosphate. NF= no fire/unburned.

|  | **DOÑANA (prescribed fire)** | | |  | **CARTAYA (prescribed fire)** | | |  | **BERMEJA LOW (wildfire)** | | |  | **BERMEJA HIGH (wildfire)** | | | |
| --- | --- | --- | --- | --- | --- | --- | --- | --- | --- | --- | --- | --- | --- | --- | --- | --- |
| **P forms (%) in**  **organic soil** | *Pre-fire* | *Post-fire* | *1 year later* |  | *Pre-fire* | *Post fire* | *1 year later* |  | *NF* | *Post-fire* | *1 year later* |  | *NF* | *Post-fire* | *1 year later* |  |
| Orthophosphate | 24 | 45 | 24 |  | 25 | 63 | 23 |  | 21 | 67 | 79 |  | 21 | 88 | 70 |  |
| P-monoesters (%) | 70 | 50 | 70 |  | 59 | 31 | 63 |  | 60 | 29 | 17 |  | 60 | 8 | 24 |  |
| Myo-inositol | 18 | 14 | 12 |  | 8 | 4 | 11 |  | 11 | 6 | 0 |  | 11 | 2 | 0 |  |
| Scyllo-inositol | 2 | 2 | 2 |  | 3 | 1 | 3 |  | 2 | 0 | 0 |  | 2 | 0 | 0 |  |
| Phosphatidic ac. | 13 | 7 | 11 |  | 12 | 6 | 14 |  | 9 | 6 | 0 |  | 9 | 2 | 0 |  |
| β-glycerol-P | 21 | 12 | 19 |  | 28 | 11 | 25 |  | 19 | 7 | 0 |  | 19 | 0 | 0 |  |
| Other P forms | 16 | 15 | 26 |  | 8 | 0 | 10 |  | 21 | 10 | - |  | 21 | 4 | - |  |
| P-diester (%) | 4 | 3 | 2 |  | 11 | 3 | 9 |  | 12 | 2 | 1 |  | 12 | 1 | 3 |  |
| Pyrophosphate | 2 | 3 | 4 |  | 5 | 3 | 5 |  | 7 | 3 | 3 |  | 7 | 3 | 3 |  |
| Organic P (%) | 74.1 | 52.4 | 72.0 |  | 70.0 | 33.7 | 72.0 |  | 72.5 | 30.5 | 18.01 |  | 72.5 | 9.09 | 26.6 |  |
| Inorganic P (%) | 25.9 | 47.6 | 27.9 |  | 30 | 66.3 | 27.9 |  | 27.5 | 69.5 | 81.9 |  | 27.5 | 90.9 | 73.4 |  |
| *Po:Pi* | 2.9 | 1.1 | 2.6 |  | 2.3 | 0.5 | 2.6 |  | 2.6 | 0.4 | 0.2 |  | 2.6 | 0.1 | 0.4 |  |
|  |  |  |  |  |  |  |  |  |  |  |  |  |  |  |  |  |
| **P forms (%)**  **in mineral soil** | *Pre-fire* | *Post-fire* | *1 year later* |  | *Pre-fire* | *Post-fire* | *1 year later* |  | *NF* | *Post-fire* | *1 year later* |  | *NF* | *Post-fire* | *1 year later* |  |
| Orthophosphate | 44 | 47 | 50 |  | 27 | 27 | 19 |  | 43 | 34 | 30 |  | 43 | 54 | 32 |  |
| P-monoesters (%) | 49 | 47 | 47 |  | 61 | 66 | 62 |  | 46 | 56 | 57 |  | 46 | 37 | 48 |  |
| Myo-inositol | 0 | 8 | 10 |  | 18 | 26 | 17 |  | 13 | 12 | 0 |  | 13 | 0 | 0 |  |
| Scyllo-inositol | 4 | 0 | 1 |  | 8 | 7 | 5 |  | 2 | 3 | 0 |  | 2 | 0 | 0 |  |
| Phosphatidic ac. | 14 | 6 | 0 |  | 13 | 15 | 11 |  | 10 | 14 | 0 |  | 10 | 0 | 0 |  |
| β-glycerol-P | 9 | 7 | 8 |  | 14 | 8 | 19 |  | 11 | 12 | 0 |  | 11 | 0 | 0 |  |
| Other P forms | 22 | 26 | 28 |  | 8 | 10 | 10 |  | 10 | 15 | - |  | 10 | - | - |  |
| P-diester (%) | 4 | 4 | 1 |  | 11 | 5 | 13 |  | 9 | 6 | 10 |  | 9 | 4 | 11 |  |
| Pyrophosphate | 4 | 2 | 2 |  | 2 | 2 | 6 |  | 2 | 4 | 4 |  | 2 | 6 | 10 |  |
| Organic P (%) | 52.69 | 51.26 | 47.83 |  | 71.15 | 70.75 | 75 |  | 54.88 | 62.38 | 66.67 |  | 54.88 | 40.38 | 58.54 |  |
| Inorganic P (%) | 47.31 | 48.74 | 52.17 |  | 28.85 | 29.24 | 25 |  | 45.12 | 37.62 | 33.33 |  | 45.12 | 59.61 | 41.46 |  |
| *Po:Pi* | 1.1 | 1.1 | 0.9 |  | 2.5 | 2.4 | 3.0 |  | 1.2 | 1.7 | 2.0 |  | 1.2 | 0.7 | 1.4 |  |
